# Factors Influencing Efficacy of Peripheral Corneal Relaxing Incisions during Cataract Surgery

**DOI:** 10.1155/2015/706508

**Published:** 2015-06-25

**Authors:** Nino Hirnschall, Jörg Wiesinger, Petra Draschl, Oliver Findl

**Affiliations:** ^1^Vienna Institute for Research in Ocular Surgery (VIROS), A Karl Landsteiner Institute, Hanusch Hospital, 1140 Vienna, Austria; ^2^Moorfields Eye Hospital NHS Foundation Trust, London EC1V 2PD, UK

## Abstract

*Purpose*. To evaluate influencing factors on the residual astigmatism after performing peripheral corneal relaxing incisions (PCRIs) during cataract surgery. *Methods*. This prospective study included patients who were scheduled for cataract surgery with PCRIs. Optical biometry (IOLMaster 500, Carl Zeiss Meditec AG, Germany) was taken preoperatively, 1 week, 4 months, and 1 year postoperatively. Additionally, corneal topography (Atlas model 9000, Carl Zeiss Meditec AG, Germany), ORA (Ocular Response Analyzer, Reichert Ophthalmic Instruments, USA), and autorefraction (Autorefractometer RM 8800 Topcon) were performed postoperatively. *Results*. Mean age of the study population (*n* = 74) was 73.5 years (±9.3; range: 53 to 90) and mean corneal astigmatism preoperatively was −1.82 D (±0.59; 1.00 to 4.50). Mean corneal astigmatism was reduced to 1.14 D (±0.67; 0.11 to 3.89) 4 months postoperatively. A partial least squares regression showed that a high eccentricity of the cornea, a large deviation between keratometry and topography, and a high preoperative astigmatism resulted in a larger postoperative error concerning astigmatism. *Conclusions*. PCRI causes a reduction of preoperative astigmatism, though the prediction is difficult but several factors were found to be a relevant source of error.

## 1. Introduction

Patient expectations concerning unaided visual acuity after cataract surgery have increased in recent years, especially since the introduction of astigmatism correcting methods.

Peripheral corneal relaxing incisions (PCRIs), also referred to as limbal relaxing incisions, have been used for decades to reduce preexisting corneal astigmatism in cataract patients and were shown to be effective [1, 2]. They are inexpensive and simple to perform. Several nomograms have been developed to improve predictability and clinical outcomes [3, 4]. PCRIs work by flattening the steep meridian and also having a coupling effect on the flat meridian.

The aim of this study was to evaluate the influencing factors on residual astigmatism after performing PCRIs during cataract surgery.

## 2. Material and Methods

In this observational study, consecutive cataract patients that were scheduled for cataract surgery and additional PCRIs were included. Exclusion criteria were any signs of irregular astigmatism such as forme fruste keratoconus, eyes after penetrating keratoplasty, or eyes with corneal scars. Additionally, no eyes with more than 3.0 D of preoperative corneal astigmatism were included.

Preoperatively, keratometry was measured with an autokeratometer integrated into an optical biometry device (IOLMaster 500) as well as topography (Atlas, both Carl Zeiss Meditec AG, Germany). Three measurements were performed with each device at each follow-up. In the case of low reproducibility the measurements were repeated. The median was used for further analysis. Calculations for the PCRIs were performed with the Donnenfeld nomogram on an online calculator and using the autokeratometry readings (lricalculator.com). IOL power calculation was calculated using the SRK/T and target astigmatism was defined as the expected remaining astigmatism.

Prior to surgery, the horizontal meridian of the cornea was marked in the sitting position at the slit lamp. Using an insulin needle, small superficial incisions were made at the limbus in the 3 and 9 o'clock positions. Care was taken to centre the slit beam on the centre of the pupil for alignment [5]. Methylene blue colour was added to the 2 small incisions to highlight them for easier recognition intraoperatively. Finally, the correct position of the markings was verified by the observer at the slit lamp. If one of the markings was off axis, this was recorded on the case report form to inform the surgeon when positioning the corneal marker intraoperatively.

Surgery was performed under topical anaesthesia in all cases by one experienced surgeon (Oliver Findl). After a Mendez style corneal marking ring was aligned to the 2 preoperative markings, blue pen dots were made on the planned meridian. A self-sealing incision with 2.4 mm single-bevelled steel blade was performed in every study eye. An incision on the step meridian was preferred combined with one opposite PCRI. In cases where a clear corneal incision on the steep meridian was awkward, such as superonasal incisions in deep set eyes, a temporal incision was and two PCRIs were made according to the Donnenfeld nomogram.

The incision was followed by the injection of an ophthalmic viscoelastic device (OVD), capsulorhexis, phacoemulsification, irrigation/aspiration of cortical material, and injection of a cohesive OVD into the capsular bag as standard procedure. The IOL was implanted into the capsular bag. Then the OVD was aspirated thoroughly using a bimanual I/A set. Care was taken to completely remove OVD from behind the IOL by slightly displacing and tilting it and reaching behind the optic with the aspiration cannula.

In all cases, PCRIs were performed at the end of surgery using a 600-micron guided steel blade (BD Atomic Edge Accurate Depth Knife, 600 microns).

All cases received an intracameral injection of 1 mg/0.1 mL cefuroxime at the end of surgery, and a standard topical regimen was followed with bromfenac (Yellox 0.9 mg/mL, Croma-Pharma GmbH, Austria) twice daily for 4 weeks.

Keratometry and topography measurements were repeated 4 months and 1 year after cataract surgery.

Additionally, subjective refraction was performed using trial frames and the Jackson cross cylinder method and ETDRS charts (Precision Vision, USA). Additionally, an Optical Response Analyzer (ORA, Reichert, USA) was used to measure the corneal response factor (CRF) and corneal hysteresis (CH) at the 4-month follow-up.

## 3. Analysis

Astigmatism vector analysis was performed using Thibos's power vector notation [6].

The true axial eye length was calculated (Appendix A) and a simplified model of the cornea was used (Appendix B). For analysis, keratometry readings were used, if not stated otherwise.

Because it was not possible to directly measure the measurement error of the cornea, we used the difference vector between the astigmatisms measured with the keratometric method and topographic method (Appendix C).

For statistical analysis, Microsoft Excel 2011 version 14.2.3 for Mac (Microsoft, USA) with a XLSTAT 2012 plug-in (Addinsoft, USA) was used. For missing data, observations were excluded from analysis. Descriptive data are always shown as mean, standard deviation (SD), and range. For statistical modelling partial least squares regression (PLSR) was performed with XLSTAT 2012 [7]: variable importance for projection (VIP) measures the importance of an explanatory variable to predict the dependent variable. More relevant for clinicians are the suggested thresholds of the VIPs: a VIP between 0.8 and 1.0 means that the explanatory variable moderately influences the model and values of 1.0 or more mean that it highly influences the regression model. To evaluate the regression model, a bootstrap method was used to estimate the weighting of each explanatory variable. This method avoids the bias of the good fit of the model for the data it has been derived from. For this purpose a PLSR model was created for all eyes except one. The model is then tested in this one “missing” eye. This procedure is then repeated for each eye (in this case 79 times). The 95% confidence interval of the bootstrapping method is shown by the whiskers. If the whiskers touch or cross the origin of the *x*-axis, the explanatory variable should not be used in a prediction model. These values are shown in the beta coefficients plots. For interpretation purposes, the larger the absolute value of a coefficient, the larger the weight of the variable and if the confidence interval (whiskers) includes 0, the weighting of the variable is not significant.

## 4. Results

In total, 80 eyes of 79 patients were included. Five patients were lost to follow-up at the 4-month follow-up due to incompliance and 21 eyes of 20 patients were measured at the 12-month follow-up.

All 74 patients, who attended the 4-month follow-up, were analysed concerning short-term outcomes of PCRIs and to develop a PLSR model, but only those 20 patients, who also attended the 1-year follow-up, were used to analyse the fading effect of PCRIs.

Mean age was 73.5 years (SD: 9.3; range: 53.0 to 90) and the female to male distribution was 43 : 32. Thirty-nine right eyes and 36 left eyes were included; in 48 eyes a 920H/907C IOL (Rayner Surgical, UK) was used, in 23 eyes a ZCB00 (Abbott Medical Optics, USA) was used, and in 4 eyes another IOL was used; in 2 eyes ZA9003 (Abbott Medical Optics, USA) was implanted, in 1 eye MX60 (Bausch & Lomb, USA) was implanted, and in one eye a 646TLC (Acri-Tec, USA) was implanted.

Mean true axial eye length was 23.90 mm (SD: 1.84; range: 20.36 to 29.90). Mean corneal astigmatism measured preoperatively with autokeratometry of the optical biometry device was −1.82 D (SD: 0.59; 1.00 D to 4.50 D). In one case a preoperative corneal astigmatism of 4.5 D was included; in all other cases preoperative corneal astigmatism was below 3.0 D. Four months after performing PCRIs, astigmatism was reduced to 1.14 D (SD: 0.67; range: 0.11 to 3.89).

**Figure 1:**
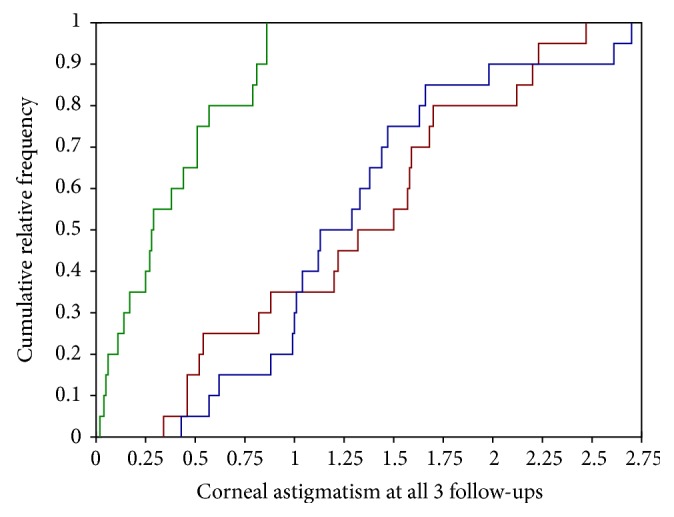
Cumulative frequency for target corneal astigmatism (green) and measured corneal astigmatism with keratometry at the 4-month (blue) and the 12-month follow-up (dark-red) in diopters.

Concerning only those patients who also attended the 12-month follow-up, preoperatively measured corneal astigmatism was −2.18 D (SD: 0.65; range: 1.32–4.53). At the 4-month and the 12-month follow-up corneal astigmatism was reduced to 1.44 D (SD: 0.85; range: 0.34 to 3.88) and 1.44 (SD: 0.82; range: 0.43 to 3.97; *p* < 0.001), respectively (Figure 1).

Using multiple pairwise comparison with a Bonferroni correction of 0.0167 a significant difference from preoperative measurements to 4-month follow-up (*p* < 0.001) and between preoperative measurements and 12-month follow-up (*p* < 0.001) was found, but not between the 4-month and the 12-month follow-ups (*p* = 0.440).

Difference vectors between the preoperative astigmatism and the measured astigmatism at the 4-month and the 12-month follow-up were found to be significant at both time-points (Wilcoxon signed rank test: *p* < 0.01; Table 1).

**Figure 2:**
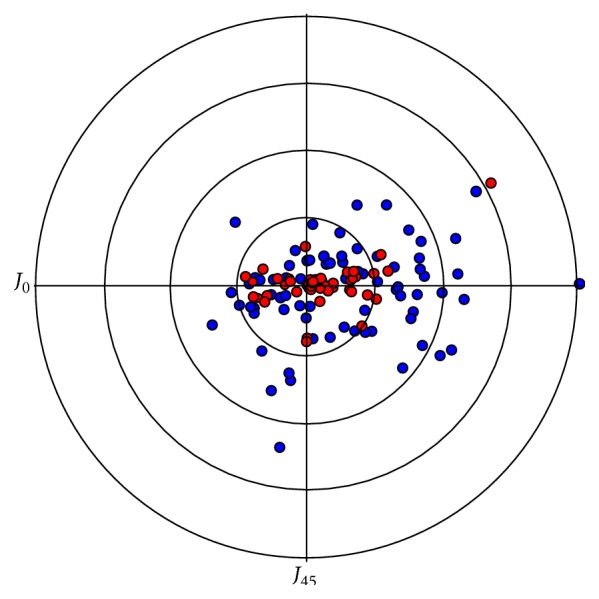
Double angle plots for aimed (red circles) and measured 4-month postoperative corneal astigmatism using a keratometry device (blue circles). Each ring represents 0.5 D.

The fading effect between the 4-month and the 12-month follow-ups was not found to be significant (Wilcoxon signed rank test: *p* = 0.501). The mean target astigmatism was 0.34 D (SD: 0.47; range: 0.34 to 2.98). Mean difference vector between this target astigmatism and the measured astigmatism at 4 months and 12 months was found to be significant at both follow-ups (Wilcoxon signed rank test: *p* < 0.01) (Figure 2 and Table 1).

Difference between different IOL types was not found to be significant (Kruskal-Wallis one-way ANOVA: *p* = 0.756).

Mean eccentricity of the cornea measured with the topography device was 0.55 (SD: 0.11; range: 0.4 to 0.88). Corneal hysteresis and corneal response factor were 9.16 (SD: 1.87; range: 3.60 to 11.93) and 8.80 (SD: 1.91; range: 5.07 to 11.77), respectively.

Mean astigmatism of subjective refraction at the 4-month follow-up was −0.88 D (SD: 0.59; range: −3.0 to 0.0), respectively. The spherical equivalents at the 4-month follow-up were −0.27 D (SD: 0.66; range: −1.5 to 2.0) and −0.44 D (SD: 0.91; range: −3.13 to 0.88 D), respectively.

Concerning only those patients who also attended the 12-month follow-up astigmatism measured with subjective refraction at the 4-month and 12-month follow-up was 1.28 D (SD: 0.94; range: 0.5 to 3.5) and 1.44 (SD: 0.86; range: 0.75 to 3.5), respectively. This difference was not found to be significant (*p*_(Wilcoxon  signed  rank  test)_ = 0.12). Spherical equivalent changed from −1.73 (SD: 1.21; range: −4.5 to −0.75) to −1.88 (SD: 1.18; range: −4.5 to −0.88), respectively. This difference was not found to be significant (*p*_(Wilcoxon  signed  rank  test)_ = 0.143).

**Figure 3:**
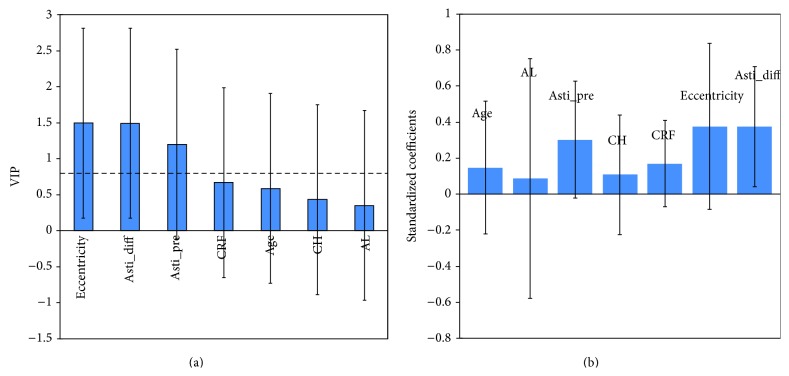
(a) Variable importance for projection (VIP) for different parameters. A value above 0.8 has an impact on the prediction of residual astigmatism; a value above 1.0 has a high impact (VIP = variable importance for projection; eccentricity = eccentricity of the cornea with the topography device; asti_diff = difference vector in corneal astigmatism between the keratometry and the topography in diopters; asti_pre = preoperatively measured corneal astigmatism (keratometry); CRF = corneal response factor; CH = corneal hysteresis; AL = true axial eye length). (b) Bootstrapping method of the PLSR model. It is shown that the model is only valid for the parameter “asti_diff”, which is the difference vector between the topography and the keratometry measurement.

A PLSR model was developed to detect those factors that had a significant impact on the deviation between the aimed and the measured corneal astigmatisms (Figure 3(a)).

A high eccentricity of the cornea resulted in a larger postoperative error concerning astigmatism, a large difference between keratometry and topography, and a high preoperative astigmatism. CRF, age, CH, and axial eye length did not show to have a relevant impact. However, in the bootstrapping model the predictive power was only significant for the difference vector of the keratometry and the topography (Figure 3(b)).

**Figure 4:**
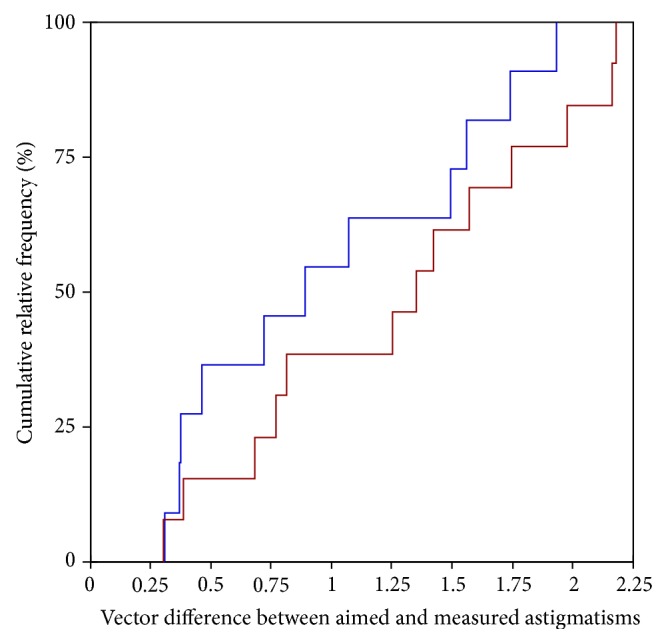
Cumulative frequency for the difference vector between the aimed and the measured corneal astigmatisms for different eccentricities of the cornea (blue = low eccentricity and red = high eccentricity).

In a next analysis step all cases were equally allocated according to their eccentricity of the cornea in two groups (eccentricity ≤ 0.52 versus eccentricity > 0.52). The cut-off value was defined by the median of the eccentricity of the cornea in the study population. Although patients with a lower eccentricity of the cornea showed a lower deviation from the aimed astigmatism (0.99; SD: 0.60; range: 0.30 to 1.93) compared to those corneas with a higher eccentricity (1.28; SD: 0.64; range: 0.31 to 2.18), this difference was not found to be significant (Wilcoxon signed rank test: *p* = 0.303) (Figure 4).

## 5. Discussion

This study investigated the potential sources of error resulting in residual astigmatism after performing PCRIs. Although residual astigmatism was reduced after performing PCRIs, a significant difference vector between the aimed and the measured corneal astigmatisms was observed. Similar findings were also reported by Mingo-Botín et al. [8]. Residual refractive astigmatism was less than 1.0 D in their study in 40% after performing a PCRI. The slight difference between both studies could be explained by the fact that different PCRI nomograms were used or by the fact that preoperative corneal astigmatism was lower in our study. These findings are contrary to observations by Poll et al. [9]. Their study mainly focused on the difference of the astigmatism vector distance of the preoperative and postoperative astigmatism but no significance levels and little explanation for the vector analysis was given. Reason could be different PCRI nomograms or different incision techniques. The method used in this study is common practice in many cataracts units. However, there is one drawback that in some cases the corneal thickness at the limbus may be thicker than expected. In these cases the 600 *μ*m guided steel blade is not cutting deep enough and the effect of the PCRI could be reduced. Astigmatism vector reduction 4 months postoperatively was similar to findings by Kaufmann et al. [10] (1.1 D after 6 months) and Budak et al. [11] (1.47 D after one month).

In cases with low preoperative corneal astigmatism the results were less predictable compared to more severe cases (Figure 3(a)). This is most likely due to the imprecise preoperative measurement of the cornea (Figure 3(a)). As shown recently, corneal astigmatism of 1.0 D is on median measured 9° off the real steep meridian, whereas higher corneal astigmatism is measured much more precisely [12, 13]. These findings were also confirmed by Shammas and Hoffer [14]. Norrby showed that 5% of all corneas show more than 0.5 D of fluctuations between measurements at different (postoperative) time-points. Although there is no evidence based explanation for this observation, diurnal changes [15], temperature, and humidity that potentially influence the tear film, pupil size [16], and asphericity of the cornea could be considered as relevant factors [17]. Furthermore, it was possible that eye drops that were instilled before the measurement could influence the measurement [18, 19].

In this study the largest source of error was the preoperative measurement of the cornea. The difference between corneal measurements was intensively discussed in the recent literature: topography and keratometry measurement devices show a good reproducibility but do not include any information about the posterior surface of the cornea. Although this is a shortcoming that results in about 0.5 D of error in one-fourth of all patients [20], no benefit was shown for IOL power calculations that include the posterior surface of the cornea measured with Scheimpflug imaging [21].

Little data is available concerning the fading effect of the astigmatism reduction of PCRIs over months after surgery. In this study it was shown that there is a fading effect of the PCRI between the 4-month and the 12-month follow-ups, but this effect is overshadowed by the deviation between the aimed and the measured corneal astigmatisms after cataract surgery. A slightly higher fading effect compared to our study has been observed by Kaufmann et al. [10] within the first 6 months after surgery. However, Kaufmann et al. only observed the fading effect within the first 6 months after surgery. Therefore, these results are difficult to compare. Similar to our study, Mingo-Botín et al. [8] observed a slight regression of the astigmatism reduction in the PCRI group. However, they only performed measurements after 3 months, whereas our study included corneal measurements 12 months after surgery.

In our study we found a large interpatient deviation of postoperative astigmatism vector reduction. Budak et al. [11] did not present their data in a similar way but observed that there was a relevant undercorrection in 75% of all cases after performing PCRI(s). Although nomograms seem to work well for the average of all patients, some corneas do not behave as predicted, possibly due to the difference in the elastic properties of the cornea and/or the extent of the scarring process after surgery. Although a direct measurement of these elastic properties of the cornea is not possible, viscoelastic properties as a surrogate parameter such as corneal hysteresis and corneal response factor could be used [22].

However, no correlation between corneal astigmatism and viscoelastic properties was found in this study and these results are in line with previous findings [23].

In contrast, eccentricity of the cornea was found to have an impact on the difference vector between the aimed and the measured astigmatisms. This finding corresponds well with findings by Park et al. [24], who observed a significant correlation between eccentricity of the cornea and surgically induced astigmatism.

In conclusion, PCRIs reduce corneal astigmatism to some extent, but the prediction of the residual astigmatism is difficult and a fading effect of the PCRIs was observed. Several factors such as eccentricity of the cornea and preoperatively measured corneal astigmatism were found to be a relevant source of error for residual astigmatism, but a significant impact was found for the difference vector between preoperative keratometry and topography measurements only.

## Figures and Tables

**Table 1:** Difference vectors in diopters (D) are shown for preoperative and aimed corneal astigmatism assessed with keratometry.

Difference vector in D	4-month group	1-year group
Preoperative	1.28 D (SD: 0.77; range 0.16 to 4.50)	1.54 (SD: 1.13; range: 0.27 to 5.16)
Target astigmatism	0.94 D (SD: 0.52; range: 0.11 to 2.18)	1.10 (SD: 0.61; range: 0.08 to 2.75)

## Appendices

### A. Calculation of the Axial Eye Length

See [25]; consider

(A.1) $$\begin{array}{c} AL=\frac{\left({AL}_{IOLMaster}*0.9571+1.3033\right)*1.3549}{1.3574}, \end{array}$$

where AL is true axial eye length and AL_IOLMaster_ is axial eye length as given by optical biometry.

### B. Calculation of the “Power” of the Cornea

Consider

(B.1) $$\begin{array}{c} K=\frac{332}{2*\left(r_{1}+r_{2}\right)}, \end{array}$$

where *K* is “power” of the cornea and *r*_1_ and *r*_2_ are radii of the cornea in mm obtained by the IOLMaster 500.

### C. Difference Vector between Topography and Keratometry Measurements

Consider

(C.1) $$\begin{array}{c} dV=2\sqrt{{\left(J_{0}ker-J_{0}top\right)}^{2}+{\left(J_{45}ker-J_{45}top\right)}^{2}}, \end{array}$$

where dV is distance vector between two measurements, ker is keratometry, and top is topography.
